# Usability and consistency of harm information in drug product descriptions: a matched comparison of data between the United States (US) and Europe

**DOI:** 10.1186/1745-6215-16-S1-P17

**Published:** 2015-05-29

**Authors:** Victoria R Cornelius, Kun Liu, Janet Peacock, Odile Sauzet

**Affiliations:** 1 Dept Primary Care and Public Health Sciences, King's College London, London, SE1 3QD UK; 2 AG Epidemiology and International Public Health, University of Bielefeld, D-33501 Bielefeld, Germany

## Background

Good information on the harm of a drug is vital to inform risk-benefit decisions and undertake robust cost effectiveness analysis. Clinical trials reported in peer-reviewed articles are not useful for this purpose [1,2]. Regulators require pharmaceutical companies to produce product information documents (Europe:SmPC, US:USPI). These documents contain comprehensive and valuable publicly available information on the known harm of a drug and have the potential to inform important risk-benefit decisions. We reviewed the usefulness of the data presented and compared the harm profile reported in documents for brand drugs marketed in Europe and the US.

## Method

Inclusion: Antidepressants/antiepileptic brand drugs evaluated in randomised trials of neuropathic pain and marketed in the US and Europe. Documentation was obtained from the European Medicines Agency and Food Drug Agency.

## Results

**Figure 1:**
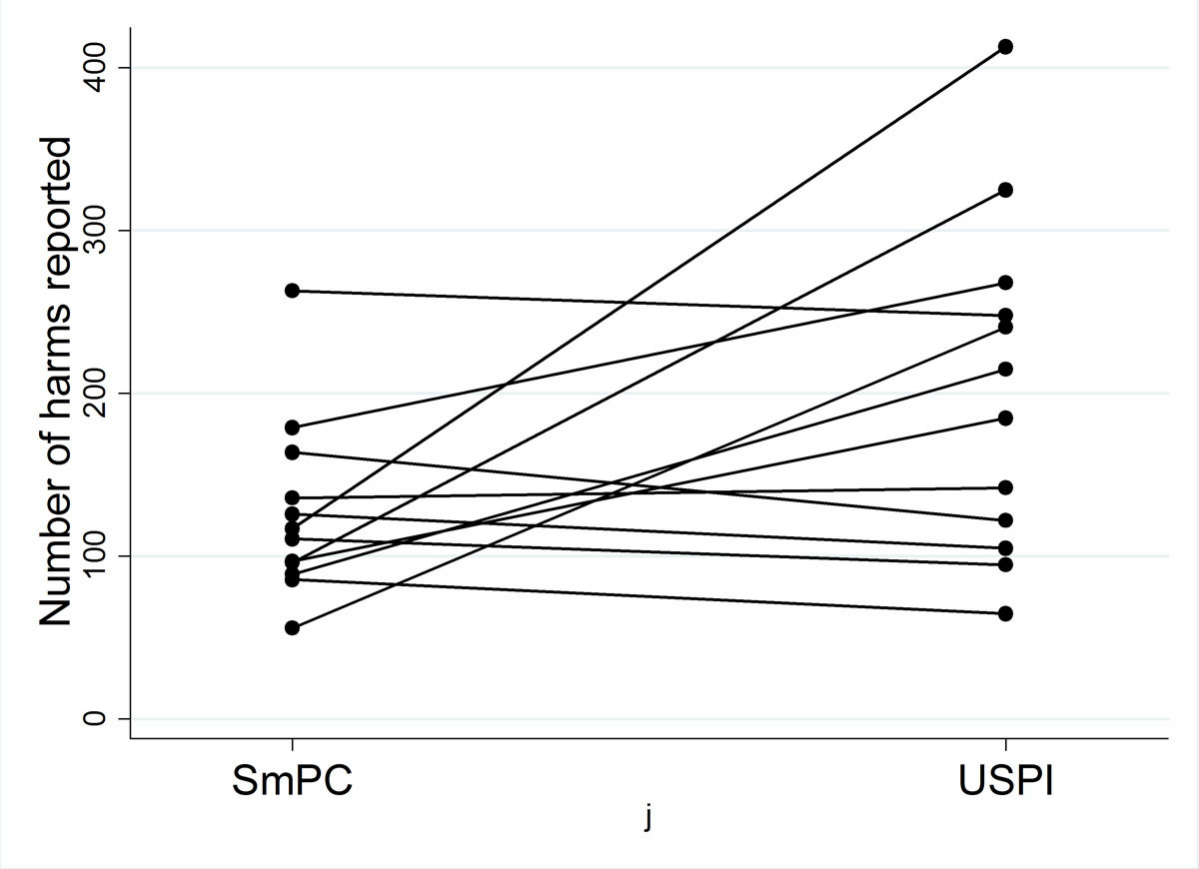
Number of harms reported by matched document

Twelve brand drugs with matching SmPC and USPI were included. The number of harms ranged from 56 to 265 for SmPC and 65 to 425 for USPI (Table 1). On average the USPI contained 70 more harms than the SmPC (Figure 1). Large numbers of AEs are collected during clinical trials, the criterion for selecting harms to report was seldom clear. Twelve of the 24 documents did report selection criteria but for 8 of these the criteria varied within the document. Medical terminology dictionaries are used to code AEs with an aim to standardised reports. More USPIs than SmPCs specified the dictionary used (6/12 v 3/12). No matched documents reported using the same dictionaries.

**Table 1:** Summary of number of harms by document

	Median # harms reported (range)		Median # harms reported **only in one document**(range)		Median # median # of the same harms reported by both documents (range)	
SmPC	114	(56, 265)	43	(20, 181)	75	(36, 104)
		
USPI	200	(65, 425)	168	(38, 336)		

## Discussion

It is expected that the harm profile in the product information for the same drug should agree. This study found a lack of consistency for the same drug based on the same central data available to the pharmaceutical company, and demonstrates the overwhelming impact of using arbitrary rules for reporting and differing dictionaries to code harm data. This problem can only be exacerbated across drugs.

The development of CORE harm outcome sets by drug class would improve the usability of this information by facilitating comparison of harm profiles across drug which would support informed risk-benefit decisions and allow robust cost effectiveness analyses.
